# A Comprehensive Review of Epidemiology, Risk Factors, Clinical Manifestations, Antifungal Susceptibility, and Mortality of Candida auris in Saudi Arabia

**DOI:** 10.7759/cureus.89455

**Published:** 2025-08-05

**Authors:** Ali Almajid, Adel Zeidan, Fatimah Alsaihati, Khaled Alghashmari, Zainab Almousa, Tala Ashour, Shahad Almarwani, Fatimah Alqahtani, Manar Alqahtani, Yamama Aljishi

**Affiliations:** 1 Internal Medicine, King Fahad Specialist Hospital, Dammam, SAU; 2 College of Medicine, Imam Abdulrahman Bin Faisal University, Dammam, SAU; 3 Internal Medicine, King Abdulaziz Specialist Hospital, Taif, SAU; 4 College of Medicine, Dar Al Uloom University, Riyadh, SAU; 5 College of Medicine, Umm Al-Qura University, Makkah, SAU; 6 College of Medicine, Vision College, Riyadh, SAU; 7 Medicine, Najran University, Najran, SAU; 8 College of Medicine, King Khalid University, Abha, SAU

**Keywords:** candida auris, candidemia, echinocandins, risk factors, saudi arabia, susceptibility

## Abstract

Candidemia, a common hospital-acquired bloodstream infection, is associated with significant mortality, particularly in cases involving *Candida auris* (*C. auris*). The Middle East, including Saudi Arabia, has seen an increasing number of invasive *C. auris* infections. This review examines the epidemiology, risk factors, antifungal susceptibility, clinical manifestations, and mortality associated with *C. auris*, based on published literature from Saudi Arabia. In June 2024, a literature search was conducted using PubMed, Web of Science, and Google Scholar to identify studies on *C. auris* in Saudi Arabia. Relevant studies were selected based on title and full-text evaluations. Inclusion criteria focused on primary research conducted in Saudi Arabia that addressed the prevalence of *C. auris*, risk factors, colonization, infections, and mortality. A pooled analysis was performed to assess the prevalence, risk factors, and antifungal susceptibility. The mortality analysis included only studies that explicitly reported mortality as an outcome. Antifungal susceptibility analysis included all isolates from primary research, based on the United States Centers for Disease Control and Prevention (CDC) minimum inhibitory concentration (MIC) cut-offs or explicit reports of susceptibility. A total of 12 observational studies comprising 315 patients were analyzed. The mean age was 63 years, with a weighted mean of 37.54; 66% (8/12) of studies reported a mean age over 60 years. *C. auris* infection and colonization demonstrated a notable gender disparity. The most common risk factors included intensive care unit (ICU) stay, recent antimicrobial use, presence of central venous catheters, and mechanical ventilation. In infected individuals, the bloodstream was the most frequent site, followed by urine. Crude mortality was 46.8% (118/252), with 80.5% (95/118) of deaths occurring within 30 days. This review highlights a high prevalence of *C. auris* among older patients, with a significant male predominance in Saudi Arabia. Candidemia was the most frequent clinical presentation. Echinocandins continue to show the highest susceptibility, making them the most appropriate empirical antifungal agents when *C. auris* fungemia is suspected.

## Introduction and background

Infections caused by *C. auris* carry a mortality rate of approximately 38%, compared to 51% for infections caused by other Candida species [1]. Candidemia remains one of the most common hospital-acquired bloodstream infections, with *Candida auris* emerging as a particularly concerning pathogen due to its high morbidity and mortality rates. *C. auris* was first identified in 2009, isolated from the ear canal of a patient in Japan [2]. Since its discovery, it has gained global recognition as a serious public health threat. Numerous outbreaks have been reported worldwide, including in the United States, Canada, the United Kingdom, Spain, India, Pakistan, Russia, Kenya, South Africa, and Colombia [3].

By early 2021, cases had been documented in 47 countries, with presentations ranging from isolated infections to widespread outbreaks [4]. The Middle East has followed similar trends, with confirmed cases in Kuwait, Israel, Oman, the UAE, Iran, Qatar, and Saudi Arabia [5]. In Saudi Arabia, *C. auris* was first detected in 2017 from blood and pleural tissue samples at tertiary care hospitals in Dammam and Riyadh [6]. Since then, several reports and case series have described hospital-based outbreaks, underscoring the increasing clinical and epidemiological relevance of *C. auris* in the Kingdom [7-17].

Given the rising incidence of *C. auris* and its potential for healthcare-associated outbreaks, this review aimed to present an in-depth analysis of *C. auris* in Saudi Arabia. It focuses on epidemiology, risk factors, clinical features, antifungal susceptibility, and associated mortality, while also addressing implications for infection control, public health policy, and regional clinical management.

## Review

Method

A comprehensive literature search was conducted in June 2024 using PubMed, Web of Science, and Google Scholar to identify relevant studies on Candida auris in Saudi Arabia. The search strategy included the following terms: PubMed - (“Candida auris”[MeSH Terms] OR “*Candida auris*”[All Fields]) AND (“Saudi Arabia”[MeSH Terms] OR “Saudi Arabia”[All Fields]); Google Scholar - “*Candida auris*” AND “Saudi Arabia”; and Web of Science - Topic: (“*Candida auris*” AND “Saudi Arabia”) OR (“*Candida auris*” AND “KSA”). The initial search yielded 886 citations. Due to limitations in relevance ranking within Google Scholar, only the first 200 results were screened. Titles and abstracts were reviewed to identify potentially relevant studies, and the full texts of selected articles were assessed for eligibility.

Studies were included if they were conducted in Saudi Arabia or directly related to the region. Eligible studies comprised original research addressing epidemiology, colonization, infection, antifungal susceptibility, risk factors, and/or mortality associated with *C. auris* across all age groups. We collected pooled data from the included studies and conducted simple statistical analyses to estimate the prevalence and identify associated risk factors. Only primary research conducted within Saudi Arabia was included. For the mortality analysis, studies were required to explicitly report mortality outcomes. Studies that lacked data on both risk factors and mortality were excluded from the pooled evaluation.

We included antifungal susceptibilities from all reported *C. auris* isolates from eligible studies with susceptibility data. We acknowledged that multiple isolates could originate from a single patient. An isolate was considered susceptible if its minimum inhibitory concentration (MIC) met or was below the CDC-established breakpoints for *C. auris* or if susceptibility was reported by the primary study, regardless of MIC values. The following CDC MIC breakpoints were applied: fluconazole: ≤32 µg/mL, amphotericin B: ≤2 µg/mL, anidulafungin: ≤4 µg/mL, caspofungin: ≤2 µg/mL, and micafungin: ≤4 µg/mL.

Only studies explicitly reporting susceptibility data for micafungin and anidulafungin were included in calculating susceptibility percentages for these agents. Studies lacking data for these drugs were excluded from that portion of the analysis. Second-generation triazoles, such as voriconazole, were excluded due to the absence of clearly defined CDC breakpoints.

Results

We conducted a comprehensive analysis encompassing 12 observational studies, which collectively involved a total of 315 patients. The mean age is 63 years, while the weighted mean is 37.54, with most studies (66%, 8/12) reporting a mean age greater than 60 years. Table 1 summarizes the prevalence by age.

**Table 1:** Summary of the prevalence of Candida auris by age.

Design	Year	Mean age (years)	Number of cases
Case report [6]	2018	43	3
Case report [7]	2019	68	1
Case report [8]	2020	64	7
Case report [9]	2021	72	2
Case report [10]	2021	48	1
Case series [11]	2021	63	5
Outbreak investigation [12]	2022	63	7
Retrospective study [13]	2021	64	35
Retrospective study [14]	2023	58	27
Retrospective study [15]	2023	64	53
Retrospective study [16]	2023	47	129
Retrospective study [17]	2024	64	46

**Figure 1:**
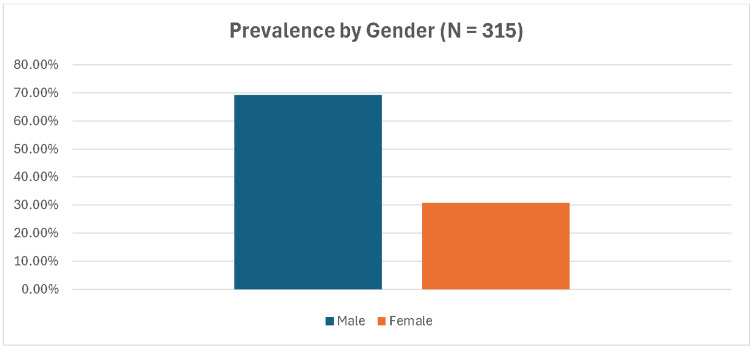
Prevalence of Candida auris by gender.

These studies exhibited significant heterogeneity in their inclusion and exclusion criteria, as well as variability in study design. Our analysis revealed a pronounced gender disparity in the prevalence of *Candida auris* occurrence. Prevalence by gender is depicted in Figure 1.

Furthermore, we identified multiple predominant risk factors associated with *Candida auris* acquisition. The four top factors were as follows: ICU stay, recent use of antimicrobial agents, central venous catheters, and mechanical ventilation at the time of *Candida auris* identification. Table 2 presents other risk factors associated with *C. auris*.

**Table 2:** Risk factors for Candida auris acquisition. DWCA: diagnosed with *Candida auris*

Risk factor	Prevalence (N=315)
ICU	72.26%
Recent antimicrobial	62.58%
The central line uses	51.29%
Mechanical ventilation	44.52%
Urinary catheter use	42.58%
Diabetes mellitus	26.77%
Hypertension	23.55%
Heart disease	18.71%
renal impairment	17.10%
Immunosuppressive drugs	15.48%
Recent surgery	14.84%
Indwelling device	14.19%
Total parenteral nutrition	10.65%
COVID-19	13.55%
Recent hospitalization	12.90%
Lung disease	11.29%
Trauma	9.68%
Blood transfusion	4.19%
Chronic liver disease	4.19%
Oncology	4.19%
Burns	2.58%
Staying with a patient DWCA	2.26%
Endoscopy	1.29%
HIV	0.65%

Additionally, our findings revealed 118 mortality cases out of a total of 252, resulting in a crude all-cause mortality rate of 46.8% (118/252), with 80.5% (95/118) of deaths occurring within 30 days. A total of 90 *C. auris* isolates were screened for antifungal susceptibility across the included studies. Of these, 95.5% (86/90) are susceptible to caspofungin. A similar susceptibility rate was observed for anidulafungin: 96.4% (55/57), and micafungin: 96.3% (53/55). In contrast, susceptibility to amphotericin B was observed in 76.6% (69/90) of *C. auris* isolates, while only 14.4% (13/90) exhibited susceptibility to fluconazole. Table 3 summarizes susceptibility patterns.

**Table 3:** Summary of antifungal susceptibility of Candida auris. NA: not available

Studies	Fluconazole	Micafungin	Caspofungin	Anidulafungin	Amphotericin B	Total isolates (N=90)
Abdalhamid et al. [6]	0	3	3	3	1	3
Almaghrabi et al. [8]	1	NA	7	8	3	8
Aljindan et al. [11]	0	6	6	NA	3	6
Kaki et al. [14]	11	NA	26	NA	24	27
Munshi et al. [17]	1	44	44	44	38	46

Table 4 summarizes the incidence of *Candida auris* by infectious syndrome and colonization versus infection status. Among a total of 315 patients, candidemia was the most common condition, affecting 31.1% (98 patients). A smaller proportion, 3.8% (12 patients), had other infections, including intra-abdominal infections, surgical stump infections, and empyema.

**Table 4:** Summary of the most common clinical manifestation of Candida auris.

Category	Incidence (N=315)	Percentage
Blood (candidemia)	98	31.1%
Urine	21	6.7%
Others	12	3.8%

Discussion

*C. auris* is an emerging pathogen of global concern, with mortality rates reported as high as 66% [14]. This review aimed to explore the epidemiology, clinical characteristics, risk factors, antifungal resistance, and outcomes of *C. auris* occurrence in Saudi Arabia, conducting a pooled descriptive analysis of published data.

Prevalence by Age and Gender

In Saudi Arabia, the prevalence of *C. auris* is notably associated with age. A retrospective study analyzing 53 patients revealed that the incidence of *C. auris* was significantly higher among individuals aged 51 years or older (79.2%) compared to those aged 50 years or younger (20.8%) [15]. Furthermore, the study found a progressive increase in incidence with advancing age, showing an approximate 2% rise for every decade above 51 years of age. This finding is consistent with other studies, which indicate that nearly half of cases occur in patients aged approximately 70 years [18].

Our analysis of 12 included studies showed that approximately 75% reported a mean age above 50 years. However, the overall weighted mean age was calculated to be 37.54 years, influenced by a study by Amer et al., which included 129 patients with a mean age of 47 years [16]. Additionally, advanced age was significantly associated with higher mortality rates in a univariate model (p=0.0038) [16].

In a meta-analysis conducted by Vaseghi et al., which included 10 studies and a total of 1,942 patients, the prevalence of *Candida auris* infection among men was found to be 80%, with an odds ratio of 3.2, indicating that men are three times more likely to be infected compared to women [19]. Our findings align with this data, showing a male-to-female ratio of 70:30 among cases.

Clinical Manifestations

The clinical presentation of *C. auris* infections mirrors that of other Candida species. It has been isolated from various sites, including blood, urine, skin, wounds, lungs, ears, and the abdominal cavity [14,15]. The most common clinical syndromes include candidemia and urinary tract infection (UTI). Rare complications, such as endocarditis and endophthalmitis, have also been reported, although their prevalence may be underreported due to limited investigations in included studies.

Risk Factors

The most frequently identified risk factors for *C. auris* infections were ICU admission, recent antimicrobial use, the presence of a central line, and mechanical ventilation. The study by Amer et al. found these risk factors to be associated with increased mortality in a univariate logistic regression model [16]. However, we identified no statistically significant associations for diabetes mellitus, hypertension, COVID-19, or kidney disease. These risk factors align with those reported in other studies [20].

Antifungal Susceptibility

In our review, five studies reported the antifungal susceptibility of *Candida auris*, involving a total of 91 isolates. These isolates were consistently tested against fluconazole, echinocandins, and amphotericin B. Echinocandins showed the highest susceptibility rate (96.4% for anidulafungin), with no discordance observed between agents when multiple echinocandins were tested simultaneously. These susceptibility rates align with globally reported figures [20].

In contrast, fluconazole exhibited a markedly lower susceptibility rate of only 14.2%. This result is consistent with the findings of Deshkar et al., who reported a 16% susceptibility rate among 50 *C. auris* isolates [21]. Additionally, other studies have documented a significantly higher resistance rate, with more than 90% of *C. auris* isolates exhibiting resistance to fluconazole [22-24]. These findings support the empirical use of echinocandins in suspected or confirmed cases of *C. auris* infection, pending susceptibility results.

Mechanisms of resistance were investigated by AlJindan et al., who examined gene mutations associated with *C. auris* in six isolates. Their analysis identified two mutations in the ERG11 gene (F132Y and K143R), which are known to confer resistance to fluconazole. No mutations were detected in the FKS1 gene, which is associated with echinocandin resistance [11].

Mortality and Treatment Outcomes

The mortality rate for invasive *C. auris* infections is substantially higher than that of other Candida species, with crude mortality rates ranging from 30% to 72% [22,25-29]. In Saudi Arabia, a study conducted in Jeddah reported a crude mortality rate of 66.7% among 27 patients diagnosed with *C. auris* infections [14]. Despite receiving appropriate antifungal therapy, including empirical treatment initiated within 24-48 h of culture results, the outcomes remained poor. This aligns with findings from other studies, where antifungal treatment, even when prolonged, did not significantly improve outcomes [28,30,31].

Mortality was particularly high among older patients with comorbidities, such as heart disease, diabetes, malignancies, or those undergoing immunosuppressive therapy. These findings underscore the need for heightened vigilance and early intervention in managing *C. auris* infections, especially in high-risk populations.

Strengths and Limitations

Our study presents several notable strengths. Firstly, it is the first comprehensive review of *Candida auris* in Saudi Arabia, providing valuable insights into the epidemiology and clinical impact of this emerging pathogen in the region. Secondly, a rigorous and well-documented methodology was employed, enhancing the reliability and reproducibility of the study's findings. Thirdly, the pooling of data from multiple observational studies enabled us to provide a broader understanding of the overall burden of *C. auris* infections and to identify common trends, thereby offering a more comprehensive perspective on the disease. However, several limitations must be acknowledged. Firstly, the study only included observational studies, with no randomized controlled trials available for inclusion, which limits the strength of causal inferences. Secondly, the included studies exhibited significant heterogeneity, encompassing case reports, retrospective studies, and cross-sectional studies, which may introduce variability and affect the robustness of the pooled data. Lastly, the analysis did not include other antifungal agents, such as triazoles, which are relevant for the treatment of *C. auris* infections. This omission may limit the comprehensiveness of the antifungal susceptibility profile presented in the study. These limitations should be considered when interpreting the results, and future research with more robust study designs and broader antifungal assessments is needed to further clarify the clinical management of *C. auris* in Saudi Arabia.

## Conclusions

This comprehensive review highlights *Candida auris* as an emerging global threat with significant clinical and epidemiological implications, particularly in Saudi Arabia. The findings emphasize the pathogen's strong association with advanced age, male predominance, and critical care settings, such as ICU admissions and mechanical ventilation. Despite echinocandins demonstrating high susceptibility rates and being recommended as first-line empirical therapy, the pathogen's high mortality rates, even with prompt and appropriate antifungal treatment, underscore its formidable nature. Further high-quality research is essential to explore strategies to enhance treatment outcomes and address the significant challenges posed by this multidrug-resistant pathogen.
